# A multi-modal recruitment strategy using social media and internet-mediated methods to recruit a multidisciplinary, international sample of clinicians to an online research study

**DOI:** 10.1371/journal.pone.0200184

**Published:** 2018-07-06

**Authors:** Cliona J. McRobert, Jonathan C. Hill, Tim Smale, Elaine M. Hay, Danielle A. van der Windt

**Affiliations:** 1 Arthritis Research UK Primary Care Centre, Research Institute for Primary Care & Health Sciences, Keele University, Keele, Staffordshire, United Kingdom; 2 School of Health and Rehabilitation, Keele University, Keele, Staffordshire, United Kingdom; Institut Català de Paleoecologia Humana i Evolució Social (IPHES), SPAIN

## Abstract

**Background:**

Challenges exist in recruiting an international sample of clinicians and researchers to an online survey. Traditional recruitment methods remain relevant but issues such as narrow geographical reach, high cost and time intensity limit what can be achieved when aiming to recruit an international, multi-disciplinary sample. Internet-mediated and social media approaches to recruitment and engagement offer new, untested ways of capitalizing upon existing professional networks.

**Objective:**

To develop, use and appraise a multi-modal recruitment strategy for an online, international survey regarding the management of shoulder pain.

**Methods:**

Traditional recruitment methods were combined with internet-mediated recruitment methods to form a multi-modal recruitment strategy. An overview of the development of this three-month recruitment strategy is provided and the value and role of each strand of the recruitment strategy discussed.

**Results:**

In response to the multi-modal recruitment strategy, data was received from 565 clinicians and researchers from 31 countries (64% UK). Complete data was received from 387 respondents with no demographic differences between respondents who completed, and those who started but did not complete the survey. Over 30% of responses were received within 1 week, 50% within 4 weeks and 81% within 8 weeks.

**Conclusions:**

This study shows the acceptability and international, multidisciplinary reach of a low cost multi-modal recruitment strategy for an online survey of international clinicians and researchers. Incorporating the use of social media proved to be an effective, time and resource-efficient recruitment strategy for this online survey and appeared to enhance clinician engagement. A multimodal recruitment strategy is worthy of consideration for future online surveys of clinicians and researchers.

## Introduction

Specific challenges exist to obtaining high response rates in surveys of clinicians, meaning that access to clinicians for research purposes can be challenging [1]. Barriers to the engagement and recruitment of clinicians in research studies commonly include lack of time, lack of interest in the research question [1–3] and, the tension for clinicians between clinical practice and participating in a study that does not reflect clinical reality, due to the constraints and limitations of empirical scientific enquiry. For data protection reasons, researchers are typically granted limited access to national and international databases of clinicians in healthcare systems or professional societies [2,3]. Therefore large-scale studies involving clinicians as research subjects are challenging.

In a separate study, we aimed to investigate how clinicians make treatment decisions for patients with shoulder pain and whether professional or international differences in decision-making exist. An international survey among a multidisciplinary sample of clinicians and academics interested in the management of shoulder pain was designed to investigate patient characteristics associated with clinicians’ choice of treatment for shoulder pain. This paper describes the design and evaluation of a multi-modal strategy that aimed to recruit a multi-disciplinary and international sample of clinicians for this survey.

A wide range of recruitment strategies were considered to decide on the optimal approach to recruitment. In order to maximise generalisability and minimise recruitment bias for a research survey, the source population from which participants are sampled should ideally replicate the target population as closely as possible. Traditional survey recruitment methods include postal invitation using mailing lists from relevant organisations, groups or societies or in-person recruitment at specific locations or events, for which the efficacy, strengths and limitations have been described in a previous review [4]. For example, a recent survey regarding the diagnosis and management of shoulder pain was conducted among general practitioners (GPs) in the UK. The survey used traditional methods to recruit GPs from an existing database, returning a 22% response rate for the paper version of the survey and 7.4% for the online version, reflecting typical response rates for both recruitment methods and also the specific challenges in recruiting health professionals to survey research [5].

In contrast to recruiting a sample of clinicians from one country and where a commercially available database of the target population exists, we aimed to recruit an international sample of healthcare professionals with a specific clinical or research interest in the management of shoulder pain across countries, for which no clear sampling frame is available. Recruiting an international sample from an unknown population gives rise to challenges including unknown population parameters that traditional recruitment approaches alone cannot fully meet.

The benefits and challenges of common traditional and internet-mediated recruitment methods for online surveys are outlined in Table 1. Traditional recruitment methods typically rely on having complete knowledge of the source population’s parameters, can have considerable time and cost implications and whilst highly targeted, may have narrow reach and little, if any impact beyond the source population. Furthermore, loss of recruitment potential due to individuals changing their contact details is a limitation of traditional methods. In addition to traditional methods, we therefore used contemporary recruitment approaches using Internet and social media in order to reach and recruit a sample for our survey.

**Table 1:** Overview of common survey recruitment approaches.

Recruitment Method	Advantages	Disadvantages
**Word of mouth**	Low effort. Low cost. Fast.	Narrow reach. Relies on access to population. Difficult to calculate response rate.
**Conferences &** **Networking events**	Access to engaged and relevant audience.	More effort. Appropriateness of invitation depends on attendee demographics on that specific occasion. Difficult to calculate response rate.
**Postal flyers**	Personal delivery of invitation in physical form to relevant individuals. Possible to calculate response rate.	Need access to postal or email address lists of relevant professionals. Moderate cost. Time intensive.
**Notice boards**	Low cost. Low effort.	Narrow reach. Difficult to calculate response rate.
**Email invitations**	Low effort. Low cost. Quick and easy to forward. Possible to calculate response rate.	Spam filters may block emails. Easy to ignore.
**Radio/Television**	Broad reach. Novelty.	High cost. High effort. Targeting of specific audience demographic or numbers difficult. Difficult to calculate response rate.
**Online message boards**	Low cost. Low effort. Novelty.	Potentially wider reach. No guarantee on audience. Difficult to calculate response rate.
**Use of local, network, professional or society** **mailing lists**	Access to large volume of relevant potential respondents. Possible to calculate response rate	Access not guaranteed due to data protection policy of each organisation. Variable cost. Limited to information held on individuals. Potential for information being out of date.
**Commercial mailing lists**	Access to large volume of relevant potential respondents. Possible to calculate response rate.	High cost. Each mailing list relevant to one country and one professional group only. Mailing lists often not specific to clinical interest within professional group.
**Personal/professional networks**	Access to relevant potential respondents. Personal/professional connection may increase response rate. Low effort. Low cost. Fast.	Reach limited only to those known to the researchers.
**Internet approaches (study adverts on professional society/interest websites)**	Moderate effort. Fast. Low cost. Likely to be viewed by relevant professionals.	No control over impact of advert.
**Social media**	Low cost. Fast. Broad potential reach. Uses existing personal/professional networks. Acceptable to approach those who are not in researcher’s network. Facilitates social sharing/snowballing. Crosses professional and geographical boundaries.	Challenging to achieve good engagement. Relies on pre-existence of a diverse and functioning social network.
**Multi-modal** **Approach**	Uses a mix of the above methods to strategically balance the pro’s and cons of each method in a multi-modal approach. Covers broader demographic and geographic areas, includes those with and without internet access. Takes advantage of pre-existing and virtual networks/groups as well facilitates opportunities offered by Internet-mediated methods e.g., social sharing amongst relevant professionals.	More time and effort required to co-ordinate plan and execute a multi-modal approach. Cost implications may exist if using software requiring licence fees or broadcasting methods that incur fees. Unable to calculate a response rate for entire approach. Cons remain of each of the methods included in the multi-modal approach.

Understanding of what the internet is has evolved from its origins of a static online space, where digital content is published and disseminated, to the emergence of ‘Web 2.0’ [6]. The concept of Web 2.0 expanded understanding of the internet to that of a dynamic and fluid space that enables participation and interaction with online content [6]. Therefore, unlike thinking of the internet purely as an online notice board, the internet offers potential to researchers for interactive, live and collaborative data collection and recruitment. A variety of internet-mediated methods for attracting research participants exist including static media such as email invitations and online message boards as well as more recently, social media and internet advertising (both generic and targeted) [7–9]. Internet-based recruitment methods are effective at reaching large, diverse pools of potential respondents, aiding generalisability whilst reducing costs compared to traditional recruitment methods [8,10–12]. Internet-based research studies also offer reduced data collection time and data entry error compared to paper surveys, can be easier, quicker and more enjoyable to complete for respondents (perceived novelty), potentially provide greater anonymity than paper surveys, and give researchers greater control over the content, timing and initial targeting of their recruitment sample [10,13–15].

Concerns prevail about potential for selection bias from internet-based study sampling. Internet-mediated research has greater potential than traditional recruitment methods to skew recruitment from certain sections of a population e.g., younger people and those regularly using the internet [16–20]. Early studies employing internet recruitment and/or data collection methods reported that their respondents were younger, more educated, predominantly white and were of a higher socioeconomic status [7,19,21,22]. However, a more recent review [10,23] identified numerous studies that have reported little differences in respondent characteristics between internet and traditional paper surveys. As use of internet and social media continues to increase globally [24], the demographics of its users also expands to represent the general population more closely.

Individuals’ lack of access to the internet is a commonly reported limitation of internet-based studies and some suggest it is an even greater issue than individuals having a lack of willingness to participate [13,15,25]. Whilst this was a valid concern in 1996, some 20 years later a report by the UK communications regulator, Ofcom [16] suggests that the use of the internet, email and social media has increased substantially over the last 10 years with 87% of the UK population now using the internet at least once weekly. Increasing from 30% in 2005, 73% of UK adults in 2016 were internet users with at least one social media account, of whom 65% access social media daily. Although younger people (aged 16–24 years) have traditionally been and remain the highest users of social media, adults in all other age categories show markedly increasing use of social media [1,26]. A global upward trend in the availability of communications services exists with data communication now surpassing voice communication in both fixed and mobile networks [24].

Confirmation exists that health and medical professionals are regularly using social media and online resources for their professional interactions, individual learning, interacting and promoting existing research recruitment and the dissemination of study findings, via the Internet [8,27,28]. Over time, with anticipated greater clinician engagement online, survey response rates are similarly likely to increase and recruited samples are more likely to reflect their target population. This will reduce the risk of selection bias and increase the precision of estimates drawn from online survey samples.

Health and medical research recruitment via internet and social media platforms is increasingly prevalent [8,12,17, 29–31]. However, far fewer studies have used internet and social media platforms to recruit clinicians as research participants [18,19]. Social media may be defined as the various online platforms that enable social connections with a wide variety of people, including potential research participants [10,32]. In research, social media can facilitate social recruitment and maximise impact and distribution capacity of an existing professional network, beyond solely those known to researchers. When an individual or society view the research invitation and opt to share or ‘re-tweet’ the research invitation, endorsement or support of the research invitation is implied [17,33]. Much in the same way as traditional research relies on clarity, transparency and the reputation of the research team; social media creates the opportunity to rapidly build an online network of relevant individuals who may then be invited to act as part of the sample and also facilitate the recruitment approach. Support for the research is implied via social sharing, lateral communication that has a ‘multiplier effect’ (10,11). In snowball sampling for online surveys, an invitation is sent to researchers’ professional and personal network for redistribution as an efficient and valid approach to recruiting an unknown population [13–15].

Good uptake and response rates to online studies is reported when multi-modal methods of study advertising and recruitment are used [16–20]. Yet, in spite of increasing use of social media in research and recruitment, few studies provide insight into their exact social media or internet recruitment strategy [19,21]. Furthermore, whilst numerous studies have examined the cost and recruitment efficiency of paid-for versus cost-free online recruitment strategies in research [17,18,30], no study has yet explored the impact of a multi-modal recruitment strategy using only cost-free online recruitment methods. Therefore, the objective of this paper is to outline the development, operationalization and appraisal of a specific example of a multi-modal recruitment strategy, that aimed to recruit a specific, yet professionally and geographically diverse sample of clinicians and researchers for an online, international survey.

## Methods

This study was hosted on a University server and employed a custom-built survey platform as the study required block randomisation and within block randomisation of survey questions. At the time of conduct, no commercially available survey host offered this. The survey was designed using a responsive layout that resized the content depending on user screen size. This enabled the survey to be accessed from mobile and non-mobile devices with ease.

The study recruited potential participants over a three-month period between 18.03.15 and 18.06.15. Eligibility criteria for the survey were: being a qualified clinician (General Practitioner, Orthopaedic Surgeon, Rheumatologist, Physiotherapist of other professional) who manages shoulder pain as part of their routine clinical practice or researcher/academic with an interest in the management of shoulder pain. The recruitment target was to collect complete data from 240 participants during this 13-week period (the required sample size was calculated on the basis of planned statistical analysis of the survey data. The results of the survey, which used a conjoint analysis approach, will be presented in a future publication). A multi-modal recruitment strategy was designed to maximise the networking potential of the study team and professional networks in a co-ordinated manner to distribute and spread the survey invitations as widely as possible across professional and geographical boundaries (Table 2).

**Table 2:** Overview of survey targeted recruitment strategy.

Method	Professional Background Targeted					Country Targeted	
	General Practitioner	Physiotherapist	Orthopaedic Surgeon	Rheumatologist	Other relevant professional	UK	Non-UK
**In Person**	- Network	- Network	- Network	- Network	- Network	- Yes	- Norway - Sweden - Denmark
**Displayed Flyer**	- N/A	- Network - S.R.U.	- Network - S.R.U.	- N/A	- Network	- Yes	- N/A
**Postal Flyer**	- Network	- Network - Shoulder Units	- Network - Shoulder Units	- Network	- Network	- Yes	- N/A
**Twitter**	- Network - Societies - Individuals	- Network - Societies - Individuals	- Network - Societies - Individuals	- Network - Societies - Individuals	- N/A	- Yes	- Worldwide
**Facebook**	- N/A	- Network	- N/A	- N/A	- N/A	- Yes	- Worldwide
**LinkedIn**	- Network	- Network	- Network	- Network	- Network	- Yes	- Worldwide
**Google+**	- Network	- Network	- Network	- Network	- Network	- Yes	- Worldwide
**E-mail invitation from Study Team**	- Network	- Network	- Network	- Network	- Network	- Yes	- Worldwide
**E-mail invitation via other party**	- Societies	- Societies	- Societies	- Societies	- Societies	- Yes	- Worldwide
**Internet adverts**	- N/A	- Network - Societies	- N/A	- N/A	- N/A	- Yes	- Worldwide

Abbreviations

N/A = Not applicable

Network = Professional Network of the Research Institute for Primary Care Sciences, Keele University.

Shoulder Units = Shoulder Rehabilitation Units in the National Health Service (NHS, UK)

Societies = Professional Body/Society/Organisation relevant to professional background and clinical practice as a shoulder specialist

Individuals = Relevant individuals with Twitter accounts identified via the Hootsuite computer application

Traditional recruitment methods included:

- Flyers advertising the research survey with a web link were displayed in the Research Institute and University’s Physiotherapy & Medical Schools, and also sent to local and regional hospitals with physiotherapy and shoulder rehabilitation departments (n = 120).
- In-person survey invitations were delivered during an invited guest talk at an international conference (n = 180) and research flyers distributed at a multi-disciplinary shoulder rehabilitation training course in the UK (n = 360).
- Postal research flyer invitations were sent to professional networks (n = 1000) including local, regional and national general practice doctors, rheumatologists, orthopaedic surgeons and physiotherapists known to the study team.

Internet-mediated recruitment methods in this study included:

1. Survey invitations were distributed to the professional network of the study team and Research Institute via e-mail.
2. Study adverts were placed on websites of relevant professional bodies and special interest groups (Table 3).
3. Study adverts distributed via the electronic/email newsletters of relevant professional societies/groups (Table 3).
4. Study adverts placed on social networking websites (Twitter, Facebook, LinkedIn, and Google +) using a targeted social media strategy.

**Table 3:** Recruitment via professional bodies.

Parties Delivering the Research Invitation on behalf of the Study Team	Mode of Recruitment	Relevance to Target Population	Professional Background Targeted	Country Targeted
**British Society of Rheumatology (BSR)**	Email mailing list	Professional association	Rheumatologists	UK
**European Society for Elbow and Shoulder Rehabilitation (EUSSER)**	Email mailing List	Professional Interest Group	Physiotherapists Shoulder Surgeons	Europe
**Irish Society of Chartered Physiotherapists (ISCPT)**	Email mailing list	Professional Body	Physiotherapists	Republic of Ireland
**British Orthopaedic Association**	Email mailing list	Professional association	Shoulder Surgeons	UK
**Society for Orthopaedic Medicine (SOM)**	Email mailing list	Professional Interest Group	Physiotherapists	International
**Primary Care Rheumatology Society**	Email mailing list	Professional Interest Group	Rheumatologists General Practitioners	UK
**European League Against Rheumatism (EULAR)**	Email mailing list	Professional Interest Group	General Practitioners Physiotherapists	Europe
**Physiospot**	Online advert for study	Professional interest website	Physiotherapists	International
**Chartered Society of Physiotherapy (CSP)**	Online advert for study	Professional Body	Physiotherapist	UK

Prior to this study, members of the team were not active professional social media users but set up social media accounts specifically for recruitment purposes for this study. The professional network of the study team consisted of the informal professional (clinical and research) email contacts of the study team members and a few departmental colleagues. Examples of individuals within this network include several: members of national and international professional bodies, shoulder pain clinical interest groups, authors of randomised controlled trials in the field of shoulder pain conducted within the last 10 years, editors of journals that routinely publish research on shoulder pain, and clinicians working as clinical shoulder specialists. When contacted, network members were requested to distribute the invitation onwards through their individual networks i.e., snowball distribution of the survey invitation, where the initial distribution was targeted to those who met the eligibility criteria. The professional bodies and special clinical interest targeted for the survey (Table 3) were relevant to the topic of the survey (clinical management of shoulder pain), but exact information regarding active membership of each of these groups was not known, as the study team did not have direct access to mailing lists.

### Targeted social media strategy

Generic invitations, personal invitations and group invitations were extended via social media networking sites. All invitations specifically included the request to share and further disseminate the invitation with further personal and professional networks. Generic invitations consisted of a brief outline of the study and who was required to complete it. Posts advertising the study were placed periodically on the lead research team member’s Google+ (Google, Inc., California, USA) (eight times), and LinkedIn (LinkedIn, Co., California, USA) profile pages. Specific paid-for advertising methods on social media sites were not used in order to minimise recruitment costs. Instead a purely cost-free posting/sharing approach was employed. A specific profile page for the study named ‘Physio Shoulder Researcher’ was set up on Facebook (Facebook, Inc., California, USA). Adverts were placed on the Facebook page (eight times). On each social media platform, visitors could re-post information or updates for others in their network to view, interact with or share. Tables 2 and 3 show the variety of over-lapping traditional and recruitment methods used to recruit individuals from each professional group.

#### Optimisation of Twitter as a recruitment tool

The social media website Twitter (Twitter, Inc., San Francisco, USA) was extensively used to extend both individual personal and group invitations to participate in this research study. In total, 363 tweets were sent from the study Twitter account during the recruitment period. The majority of tweets sent contained the internet address link, requested for the invitation to be shared, and used informal and friendly language to encourage interaction and participation. The computer application Followerwonk (Moz Inc., Washington, USA) was employed to identify those who were likely to match the inclusion criteria for the survey, searching the biographical information provided on Twitter users’ profiles for the keywords: shoulder, upper limb, physiotherapy, physical therapy, medicine, doctor, general practitioner, family medicine, rheumatologist, orthopaedic surgeon. Of those identified, a list of accounts of relevant individuals, groups and societies with high ‘social capital’ was formed and these became the recruitment targets for this study. Social capital in this context was defined as the user’s social media profile having greater than 50 followers, and having posted within the last month demonstrating online interaction with individuals such as shoulder clinicians and researchers, i.e., those were likely to be potential respondents. Tweets to the targeted individuals or groups were targeted by mentioning the target’s unique username e.g., @ClionaMcR or @PCSciences, in the tweet. Followerwonk was also used to provide an indication of the most active time periods for the identified Twitter profiles. The most active times for the Twitter profiles followed by the researcher and also for followers of the researcher’s Twitter profile were 7am, 11am, 1am, 3pm, 4pm, 7pm, 9pm, and 11pm. Social media posts were scheduled to be sent during these times using the computer application Hootsuite (Hootsuite Media, Inc., Vancouver, Canada). Hootsuite was used to optimise time and resource management throughout the recruitment period and also to ensure that the research invitation featured regularly on the stream of tweets appearing on Twitter. The free versions of Followerwonk and Hootsuite were used in this study, which enabled identification of social media profiles of clinicians likely to match the inclusion criteria and scheduled posting from one social media account, that of the lead researcher.

In order to widen the international reach of the recruitment strategy, tweets were sent in multiple languages (Spanish, French, & Italian) to large international professional organisations. To make the tweets impactful on the Twitter page, pictures of shoulders, a QR code (quadratic residue code i.e., a bar code) for the study website and twitter hashtags #ShoulderResearch, #shoulder and #research were interchangeably used. Metrics from the researcher’s Twitter account during the recruitment period were obtained from Twitter Analytics and use of Twitter in the recruitment strategy evolved iteratively as the researcher monitored the level of interactions with tweets and amended the strategy as indicated.

## Results

### Observed trends in recruitment modes of access

In total, the survey was accessed 2700 times by 2326 individuals during the three-month survey recruitment period. Data was received from 1915 respondents. Data was categorized into complete data, partial data and unusable data. Complete data, defined as having provided an answer to every question on the survey was received from 387 individuals (20.2% of those who began the survey and 12.3% of those who accessed the survey). Partial data, defined as having completed at least all of the demographic data, was received from 178 individuals. Unusable data, defined as having started the survey but not completed the demographics questions, was received from 1350 individuals. Numbers of respondents who accessed the survey via the different modes of access are shown in Table 4.

**Table 4:** Respondents by survey access mode.

Access Route	No. of Respondents	% of Total Respondents	Financial Cost	Estimated Time Spent
Direct webpage link via email, flyer or verbal invitation	1029	54%	Flyer printing: £85 Postage: £636 Envelopes: £28	25 hours
Twitter	552	29%	£0	30 hours
Facebook	100	10%	£0	2 hours
Physiospot	72	4%	£0	30 min
CSP	52	3%	£0	2 hours
Google	41	2%	£0	30 min
LinkedIn	1	<1%	£0	30 min
Other online sources	68	4%	£0	30 min
**Total**	1915	100%	£749	61 hours

Data from Google Analytics (http://www.google.com/analytics) was used to determine the device categories respondents used to access the survey and also to explore the performance and impact of each of the recruitment methods (Table 5). The greatest proportion of respondents accessed the survey via a direct internet address link (n = 1029, 54%), most likely to have been gained from either a direct or snowball circulated email from the researcher or from a postal flyer that was either individually received at a conference, via postal mail or seen displayed in a hospital or university setting. Internet-mediated and social media recruitment approaches accounted for 46% of the total survey data. More than half of respondents (51%) accessed the survey on a desktop platform, 39% on a mobile phone device and 10% on a tablet. It was not possible to differentiate between these direct webpage modes of access using the Google Analytics data. Of these internet-mediated approaches, Twitter accounted for 29% of the survey data with other approaches contributing smaller proportions. Over 30% of the complete and partially complete data (n = 565) was received within the first week, 50% within four weeks and 75% within 6 weeks. The survey was closed after 13 weeks.

**Table 5:** Number of unique page views according to access device category.

Device Category	Unique Page Views	% of Total Unique Page Views
Desktop	2135	51%
Mobile	1643	39%
Tablet	437	10%
**Total**	4215	100%

Demographic details of those classified as complete responders and partial responders are presented in Table 6. Data was received from 31 different countries, which were grouped according to similarities in model of healthcare provision. The majority of respondents were physiotherapists (n = 375, 66%) and most were from the UK & Republic of Ireland (n = 263, 68%). Complete responders had more years of clinical experience (mean 16.3 versus 13.1) and more complete responders than partial responders reported that their primary clinical role was in a state-funded healthcare system (100% versus 88%).

**Table 6:** Demographics of complete and partial survey responders.

	Total Responders (n = 565)	Complete Responders (n = 387, %n)	Partial Responders (n = 178, %n)
**Professional Background**			
Physiotherapist/ Physical Therapist	371	255 (66%)	116 (66%)
General Practitioner/ Family Doctor/ Primary Care Medical Physician	75	60 (16%)	15 (8%)
Rheumatologist	36	21 (5%)	15 (8%)
Orthopaedic Surgeon	15	8 (2%)	7 (4%)
Other relevant professionals	68	43 (11%)	25 (14%)
**Country of Clinical Practice**			
UK & Republic of Ireland	352	263 (68%)	89 (50%)
Netherlands, Norway, Sweden & Denmark	67	43 (11%)	24 (13%)
Germany	3	2 (<1%)	1 (<1%)
Australia & New Zealand	28	20 (5%)	8 (5%)
USA & Canada	50	26 (7%)	24 (13%)
Rest Rest of World	65	33 (9%)	32 (18%)
**Years of clinical experience:** Mean (Std. Dev.)	565	16.31 (9.8)	13.1 (10)
**Primary clinical role in state-funded health system**	565	387 (100%)	157 (88%)

The 363 recruitment tweets were viewed on average 1400 times per day over the first 60 days of recruitment with a total of over 85000 views over the 3-month recruitment period. Tweets were shared in total 286 times via retweets, likes and on 9 instances via email. Each tweet received on average 235 views. Of the 85575 tweet views, the internet address link was accessed in 0.0065% of views (563 times).

## Discussion

### Main findings

We have provided a detailed description of a multi-modal international recruitment strategy for an online survey involving a range of health professionals and researchers interested in the management of patients with shoulder pain. The recruitment strategy was considered successful as it exceeded the aim of recruiting a multi-disciplinary and international sample of 240 participants with complete data within the defined recruitment period, with 387 complete responses received. Respondent demographics (Table 6) indicate that the multi-modal recruitment strategy enabled recruitment of a sample from a large number of countries, professional disciplines, healthcare settings and ranging experience. Given that recruitment used professional network-based snowball methods, it was not possible to calculate a response rate, however the survey access/completion rate was 20.2%. This access/completion rate is lower than the average 33% response rate in web surveys of the general public [22]. In comparison, response rate amongst health professionals appears to vary according to professional background with Bishop et al [23] reporting an average response rate of 38% in a postal cross-sectional population survey of clinicians (22% for GPs and 55% for physiotherapists), and with physiotherapists also returning a higher response rate of 58% in a more recent postal survey [1,33]. However the access/completion rate in this study is in line with the 21–26% average response rate observed in cross-sectional postal surveys of GPs [8,34].

### Strengths of the multi-modal strategy

The main strengths of the multi-modal strategy used were that barriers of geographical boundaries and international timelines were minimised, with participants successfully recruited across international borders and professional backgrounds (Table 6). Although the multi-modal approach was not empirically compared to traditional recruitment methods, the recruitment of an international sample of clinicians to the survey was possible with minimal financial and time cost (Table 4). Limited scope for international participation has been cited in the past as a weakness of web surveys [8,10,12]. However, using a multi-modal internet-mediated recruitment strategy, this study obtained response data from 31 different countries over a three-month period. The combination of multi-modal recruitment strategies delivered in parallel and in sequence resulted in a high level of engagement with the survey; with over 30% of survey data received within the first week. This indicates that the multi-modal research strategy delivered on its potential to rapidly engage and direct an unknown, professionally diverse and geographically spread target population to an online survey. Furthermore, the observation that nine individuals shared a tweet sent by the researcher via email indicates that the target population do indeed use multiple forms of communication including social media to collate and share information with peers. These emails represent multi-modal recruitment snowballing in action; an occurrence that itself generates momentum in sharing a research invitation with further, potentially untapped pockets of the target population. It cannot be known how many other such snowballs were generated via the multi-modal approaches taken to recruitment in this survey, but this observation evidences the connectedness of everyday health and research professionals and also their willingness to participate in social sharing of research recruitment invitations.

Use of internet-mediated and social media methods to recruit health professionals to research studies is relatively new [10,19]. Use of existing computer applications such as Followerwonk and Hootsuite facilitated time-efficient identification of key individuals who were invited to act as new ‘snowballs’ for recruitment of their professional network. One example from this study is the tweet sent to a physiotherapist with a clinical interest in shoulder pain, whose Twitter account had over 30,000 followers. Followerwonk identified this individual as a key recruitment target and this single tweet sent from the researchers account reached over 5000 individuals internationally with an interest in shoulder pain, a reach that would have been almost impossible relying on just the professional network of the study team and prior to the advent of social media. At least 46% of the respondent data can be directly attributed to recruitment using internet-mediated methods including social media. Use of Twitter to engage with and recruit health professionals and researchers in a research survey had little precedence at the time of designing the survey (early 2015) and use of a researcher’s personal Twitter account to recruit individuals to an international research survey was considered relatively novel. Since the lead researcher’s professional background and affiliations were clearly outlined on all internet platforms employed, as well as in all of the recruitment material, it was intended that such transparency would make it easy to judge credibility of the researcher, the study team, and the study itself.

Use of ‘broadcast’ methods as recruitment tool has been previously outlined [8, 17], whereby researchers pay social media companies to display study adverts on the timeline/live feed of individuals who meet the inclusion criteria. Weaknesses of broadcasting approaches include cost per individual, the degree to which ‘adverts’ are ignored or mistrusted on otherwise free to use social media platforms, and that broadcasting relies on an individual being online during a specific time-period that the researcher has paid the platform to broadcast within. In comparison, this study used only cost-free posts on social media pages to stimulate peer-led, socially shared, snowballing methods amongst clinical and research colleagues such as those outlined. This was hypothesised by the study team to have greater credibility and impact as a recruitment strategy and has the advantage of being cost-free. This targeted approach also has the advantage of being specifically targeted to individuals likely to meet survey inclusion criteria. This may have helped to increase the response rate as well as generate a powerful snowballing recruitment effect amongst other professionals who were unknown to the study team or perhaps not included in the international professional societies targeted. However, it is accepted that individuals, societies or groups may have been missed as the strategy relied on individuals including their specific professional interests and social media biography.

Although the online survey was provided exclusively in English, the recruitment strategy included a number of steps intended to specifically include and invite international participation. These included contacts with relevant professional bodies, societies and organisations across the world, and tweets translated by native speakers into Spanish, French and Italian. The online nature of the study also enabled participants to respond in a time that best suited them and the ‘save and return later’ facility enabled busy clinicians and researchers to fit in completing the survey between tasks or duties. Use of a web survey over a paper survey in this study facilitated immediate receipt of responses to a secure database and also allowed respondents to participate in the survey using the electronic medium of their choice (desk-top computer, mobile phone or tablet). Although 51% of respondents accessed the survey on a desktop computer, mobile phones (39%) and tablets (10%) were also used to access the survey. This highlights the necessity for future researchers to ensure that research surveys are both compatible and formatted appropriately to operate on multiple media simultaneously.

### Weaknesses of the multi-modal strategy

Recruitment, retention and representativeness are as much a challenge in an internet-mediated research study as in any other research. A significant obstacle for this study was in defining a strategy to attract and recruit an unknown population. There is an unavoidable degree of self-selection bias in any survey, where certain individuals are more likely to respond to surveys than others [17,35]. However, in the case of an unknown population, it is more difficult to assess the risk of bias.

A criticism of using social media as a recruitment tool for research studies is that respondents recruited via social media tend to be younger than those from more traditional recruitment methods [7,17]. However, data on number of years experience was collected and shows that respondents who provided complete data had more years of clinical experience on average (mean 16.3 (SD 9.8) years) than those who provided incomplete data (mean 13.1 (SD 10.0) years).

Response rate is difficult, if not impossible, to calculate as recruitment happened in person, via postal invitation, via email and online using planned internet-mediated approaches. The difficulty with response rate calculation in this context lies in the lack of methodology for tracking what happens to research invitations once they are placed online. Whilst the Google Analytics data provided insight into how each of the social media platforms and professional websites on which an advert was placed performed, one and the same web link was used to allow access to the survey website. Therefore, it was not possible to see how the different recruitment routes compared in terms of achieving survey participation. The Twitter metrics showed how often each tweet was shared and on certain websites (PhysioSpot and Chartered Society of Physiotherapy websites) how often the webpage containing the recruitment invitation was shared. However, this study did not include a data capture method that could inform the researchers about the exact access route to the survey taken by each individual responder, making it impossible to gain insight into the access to completion rates across different recruitment or social media routes. For example, it may be possible that some social media platforms generated lower traffic to the survey than others but were more successful in generating complete versus incomplete survey data. Future online surveys should either (i) include a question to assess how participants heard about and accessed the survey or (ii) use specific variations of the study web address for each mode of recruitment and access. These steps would enable analysis of the impact of each recruitment method on generation of: (i) traffic to the survey and (ii) complete response data. Also, future researchers could consider stratifying recruitment methods over time, using one method alone for a defined period before moving on to the next. Whilst this would have the disadvantage of potentially limiting the accumulation of online presence and visibility during a defined period, it would allow researchers to quantify how many respondents came from each method and whether different recruitment methods attract respondents with different characteristics.

The sample obtained is unbalanced by geography (64% from the UK & Republic of Ireland) and professional background (66% of sample were physiotherapists). In spite of advertising the study in other languages, the survey was conducted exclusively in English due to known issues with translation and loss of culturally imbued meaning [10,36], and several recruitment approaches specifically targeted organisations (Table 3) or potential participants (e.g. distribution of flyers) in the UK. Response to the survey from GPs, orthopaedic surgeons and rheumatologists was low, with physiotherapists providing 66% of responses. The strong contribution from physiotherapists may be explained in part by the lead researcher’s professional background. Steps taken to address this potential bias included specifically identifying and targeting national and international professional interest groups for non-physiotherapists as outlined in Table 3 and targeting recruitment flyers to GPs with a special interest in musculoskeletal conditions and shoulder and upper limb orthopaedic surgeons and rheumatologists known to the study team. Potential reasons for low response rate may include perception that the research area is not of relevance to the physician’s clinical practice, that they are already too busy, or simply that they do not participate in research surveys [16,37]. Impact of participation incentives for physicians to boost response rates have been not been shown to be effective amongst GPs [1,25,34,38] and were therefore not used in this study. However since it is traditionally difficult to achieve high response rates amongst physicians [13,15,39], further research is indicated to improve participation and response rates amongst clinicians in research surveys, including the potential for using a multi-modal recruitment strategy in conjunction with commercially available databases of clinicians, accepting the cost implications of such an approach.

A further challenge for the use of internet-mediated research in general is gaining complete data. Analytic data from Twitter and some of the professional Internet websites indicated that it is relatively easy to encourage potential respondents to click on the survey web address. Data from this study shows that the survey website was accessed 2700 times, with 1916 respondents submitting some data but complete data only being received from 387 individuals. Precise reasons for providing incomplete data are not clear, but since the survey was fielded only in English individuals who accessed the survey but were not fluent in the English language may have opted to leave the survey without providing complete data. Similar challenges in retaining respondents’ levels of interest and engagement to the end of the survey have previously been reported [8,26,27,30]. Respondent anonymity and the physical distance from the researcher may be a factor as individuals are less likely to feel an obligation to the researcher to complete the survey. The same challenge may occur in paper-based surveys, however with paper-based studies people decide to either complete it or not to respond at all, resulting in fewer partially completed surveys. Despite the large proportion of incomplete data, the demographic information provided indicated that respondents who provided complete data were largely similar to those providing incomplete data, and representative of the target population.

A common concern about the use of web-based surveys is that respondents are anonymous and that the authenticity of data often cannot be confirmed [8,10,12,17, 28–30]. Although it was possible to retake the survey, all data were screened for total completion times less than five minutes. No such responses were found, indicating that on balance, the data is likely to be legitimate, given the expected completion time of 15 minutes. An additional limitation is that open, unrestricted online surveys have to accept the risk that respondents may not actually be who they say they are and even that computer programmes may have been used to create spam data or that respondents may have completed the survey multiple times to create a ‘ballot box stuffing’ effect [13,18,19].

### Ethical considerations

Ethical approval for the study was gained from Keele University ethical review panel. Issues surrounding use of a web survey and a multi-modal and Internet-mediated recruitment strategy were considered, especially the use of social media to contact potential respondents. Specific details pertinent to the acquisition of ethical approval for the study included anonymity and security of the data provided. To maintain respondents’ anonymity, non-identifiable demographic questions were kept to the minimum required to characterize the sample (professional background, number of years experience, relevant post-graduate training, country of clinical practice) and Internet protocol (IP) addresses were not collected in order to protect respondents’ anonymity. Data was stored on the physically and electronically secure, restricted access Keele University server, which is routinely backed up and which was accessible only by the study team.

## Conclusions

Using a multi-modal traditional and Internet-mediated recruitment strategy was successful in recruiting a professionally diverse international sample of health and research professionals with an interest in clinical management of shoulder pain. A social media strategy involved identification of most relevant societies, organisations and individuals and sending of targeted research invitations were via social media (Twitter, Facebook, Google+ and LinkedIn) and traditional methods. Of the 565 respondents who provided data in response to this survey, social media accounted for 46%, indicating that clinicians were happy to be contacted and recruited to the research survey via social media and internet-mediated methods as well as traditional methods. Researchers can therefore consider using a multi-modal research strategy to recruit health professionals to future online studies. Whilst acknowledging limitations of the method, this approach offers a pragmatic, easy to use strategy that can be used in future studies. A multi-modal survey recruitment proforma has been developed to assist future researchers achieve the potential offered by these low-cost recruitment methods (Table 7).

**Table 7:** Multi-modal survey recruitment proforma.

Study Title		
Study Live Period	Beginning	Ending
Target Population		
Source Population		
**Recruiting a Known Population:** - Random or convenience sampling? - Available mailing lists or existing databases		
**Traditional Recruitment Methods** - Word of mouth - Paper flyers - Postal invitations - Email - Newspaper/Magazine articles in in relevant publications - Radio/TV advert - Notice boards		
**Recruiting an Unknown Population:** - Define characteristics of your target population - How will you reach this population - Outline methods of identifying key individuals/organisations		
**Internet-Mediated Recruitment Methods** - Relevant Social Media Platforms - Relevant Websites - Relevant Newsletters/Blogs		
**Identify key individuals/groups/organisations:** - Using social media analytics software (e.g., Followerwonk or similar) - When are the key posting times for your target population?		
**Outline Detailed Plan for each social media platform:** - Twitter - Facebook - LinkedIn - Google+ - Other		
**Any visual resources needed:** - Photograph of study team/lead - researcher - QR code - Study logo (for use as email banner, social media) - Any other relevant image		
**Produce a final Multi-Modal Recruitment Plan outlining:** - Daily plan - Weekly plan - Monthly plan - Automated scheduling system (e.g., Hootsuite or similar) - Review/Iteration plan		
**Additional study invitation dissemination plans:** - Email - Postal - Noticeboards - Other		
